# Up-Regulation of Cell-Free MicroRNA-1 and MicroRNA-221-3p Levels in Patients with Myocardial Infarction Undergoing Coronary Angiography

**DOI:** 10.34172/apb.2021.081

**Published:** 2020-07-26

**Authors:** Fatemeh Mansouri, Mir hosein Seyed Mohammadzad

**Affiliations:** ^1^Department of Genetics and Immunology, Faculty of Medicine, Urmia University of Medical Sciences, Urmia, Iran.; ^2^Cellular and Molecular Research Center, Urmia University of Medical Sciences, Urmia, Iran.; ^3^Department of Cardiology, Seyedoshohada Hospital, Urmia University of Medical Sciences, Urmia, Iran.

**Keywords:** MicroRNA-1, MicroRNA-221-3p, Myocardial Infarction, Artery stenosis

## Abstract

***Purpose:*** Myocardial infarction (MI), known as a multifactorial disease, remains the predominant cause of mortality and sudden deaths annually. The current study aimed to measure the expression of microRNA-1 and microRNA-221-3p in MI patients.

***Methods:*** In the current study, 100 healthy controls (with no history of heart disease) and 200 patients with MI were selected. Patients were divided into two groups based on angiography results: normal (no significant artery stenosis) and primary percutaneous coronary intervention (primary PCI, significant artery stenosis). The levels of microRNA-1 and microRNA-221-3p were quantified using real-time quantitative polymerase chain reaction. The correlation between levels of microRNAs and the common cardiac markers were analyzed statistically.

***Results:*** In comparison to fold change, microRNA-1 elevations were 8.5-fold in normal patients and 60-fold in patients with primary PCI; while microRNA-221-3p levels were 210- fold higher in primary PCI and 31.31-fold higher in normal cases compared with the healthy controls. Receiver operating characteristic analysis showed that the area under the curve (AUC) for circulating microRNA-1 and microRNA-221 were 0.903 and 0.958 in normal patients and 0.927 and 0.985 in primary PCI patients (*p* < 0.0001), respectively. Pearson correlation (*ρ*) analysis showed that circulation of microRNA-1 correlated with serum levels of cardiac troponin I (CTnI) (*ρ* =0.24), creatinine (*ρ* =0.34), creatinine kinase-myocardial band (CK-MB) (*ρ* =0.31), and microRNA-221-3p was significantly correlated with serum levels of CTnI (*ρ* =0.6), creatinine (*ρ* =0.41), and CK-MB (*ρ* =0.37), (*P* < 0.0001).

***Conclusion:*** The study underscored the potential of microRNA-1 and microRNA-221-3p as informative biomarkers and positively correlated with artery stenosis in MI.

## Introduction

Myocardial infarction (MI) remains the most common disease in the developing world and leading cause of mortality in human.^1^ Approximately 31% of deaths are annually due to cardiovascular disease. In cardiovascular disease, MI known as heart attack is recognized as the first leading cause of sudden death in human.^2^ People from the middle age group, both genders, urban and rural populations may be affected.

In order to define the clinical practice based on etiology, five main types of MI are categorized.^3,4^ Differentiation and early recognition between different types of MI would greatly help clinicians to manage in the practice. At present, electrocardiography (pathological Q waves and ST segment elevation) and biochemical laboratory tests generally used for MI detection as well as some diagnostic procedures impose significant economic burden on patients. In addition, the manifestation and progression of acute myocardial infarction (AMI) symptoms are not straightforward and overlap between MI types in some people. For better disease management, detection of specific markers in patients before manifestation of symptoms is a major issue.^5^ Molecular tests and biomarkers offer many advantages over traditional tests such as cardiac troponin I (CTnI), creatinine, creatinine kinase-myocardial band (CK-MB) and etc.^6,7^

In many diseases, changes in circulating and differentially expression levels of microRNAs in blood and other biofluids may represent useful non-invasive biomarkers for early diagnosis and prognosis. These biomarkers can be detected in patients with no warning symptoms or signs of the disease. They have high accuracy in early diagnosis and produce better treatment outcomes.^8^ Growing evidence identifies microRNAs as biomarkers in progression, detection, and prognosis of disease.^6^ The microRNAs as small molecules are involved in gene silencing, regulating gene expression, cellular differentiation, necrosis, and apoptosis.^5^ Some microRNAs are expressed in various acute and chronic cardiovascular diseases, fibrosis, arrhythmia, and heart failure.^9^ Previous studies mainly focused on microRNAs expression in the serum of patients with MI. During the progression of the disease microRNAs concentration can provide prognostic information in patients at cardiovascular risk.^10,11^

In the current study, to determine whether microRNA-221-3p and microRNA-1 in blood can be used as useful approach for AMI, their levels were analyzed in patients. The receiver operating characteristic (ROC) curves were also drawn to evaluate the sensitivity and specificity of microRNAs. The utilization of these biomarkers helps understanding the pathophysiology of heart and the opportunity to reduce mortality and morbidity. It is used to identify the association between the microRNAs and variable stenosis of artery.

## Methods and Materials

### Sample collection

The study recruited 200 patients with AMI diagnosed based on the basic diagnostic criteria within 1-12 hours of onset from January 2017 to October 2017. Two groups of patients with AMI in the heart clinic were considered. Patients were visited by an experienced cardiologist and admitted to the Seydoshohada Hospital, affiliated to Urmia University of Medical Sciences, Urmia, Iran. All patients were selected based on clinical symptoms (chest pain or exertion dyspnea) and the result of the non-invasive tests (positive exercise test, ST elevation or Thallium scan). Patients that had chest pain and positive ETT result (exercise tolerance testing), or showed ischemia in MPI (myocardial perfusion imaging) were selected to undergo coronary angiography. MPI can show areas of the heart muscle that are not getting enough-blood flow. Also, ETT is used to determine the presence of significant coronary heart disease. Coronary angiography showed normal coronary artery in 100 patients (significant stenosis was not revealed, and they were called normal patients) and 100 patients had more than 50% coronary artery stenosis of one or more vessels (called primary PCI patients). None of the patients took heparin during coronary angiography (CAG). They took heparin 100 u/kg during the percutaneous coronary intervention (PCI). Written informed consent was obtained from all the patients in accordance with the principles of the 1975 Declaration of Helsinki, and the study protocol was approved by the Ethics Committees of Urmia University of Medical Sciences. In addition, 100 subjects with no history of MI, age above 35 years, and routine health examination were studied as healthy controls. Clinicopathological information including; age, gender, weight, smoking/hobble bobble habit, family cardiac history, and pre-cardiac histories were provided for all subjects. The patients with cardiomyopathy, congenital heart disease, hepatic failure, renal failure, hepatitis, immune deficiency disease, infection, cancer, and malignant diseases were excluded.

### RNA isolation and storage

The vein blood samples of patients and controls were collected into vacuum ethylene diamine tetra acetic acid-coated (EDTA) tubes. Sera were immediately processed by centrifugation at 4000 rpm for 10 minutes at 4°C and stored at -70°C until further use. The total RNA was quickly extracted by Hybrid-R^TM^ miRNA Kit (Geneall, Seoul, South Korea) for purification of large and small RNAs separately based on glass fiber membrane technology according to the manufacturer’s instructions. The cDNA synthesis was performed on 60 ng of microRNA from each sample using the cDNA SynthesisExiqon Kit (Vedbaek, Denmark) according to the protocol and allcDNAs were kept at -70°C until further use.

### Quantification of microRNA-1 and microRNA-221-3p by quantitative real-time PCR (qRT-PCR)

Specific quantitative real-time PCR (qRT-PCR) was set up to estimate the concentrations of microRNA-1 and microRNA-221-3p in the patient and control samples and also microRNA-423-5p as an endogenous control. The reaction components were mixed well and the real-time PCR was performed in a total volume of 10 μl containing 60 ng of cDNA, and 10 pmol of forward and reverse primers using appropriate SYBR Green PCR Kit (Exiqon, Denmark) in a 48-well real-time PCR plate (Applied Biosystems, Foster City, CA, USA). The thermal cycling conditions were as follows: 95°C for 10 minutes, 40 cycles at 95°C for 10 seconds, 60°C for 60 seconds, final extension at 72°C for 5 minutes and the cycle threshold (CT) was evaluated, followed by a melting curve analysis. Also, from these tests, CT < 40 was considered suitable to avoid inclusion of non-specific amplification and the mean threshold cycle (ΔCT) of the target gene for the two replicates by fold change method (2^-ΔΔCT^) was considered for analysis.^12^

### Determination of other cardiac markers

Troponin I, CK-MB, creatinine, and other markers were measured at the time of cardiac catheterization by an autoanalyzer (Autolab, BT 3500, Auto Analyzer Medical System, Rome, Italy) and a chemiluminescent analyzer (Architect i1000 SR, Abbott, USA); peripheral blood samples were evaluated by commercial ELISA (enzyme-linked immunosorbent assay) kits according to manufacturer’s instructions.^13^ The upper and lower limits were considered in normal reference range 0-1 ng/mL for CTnI, 0-24 U/L for CK-MB, and 0.6-1.3 mg/dL for creatinine in the Clinical Laboratory of Seyedoshohada cardiovascular medical Center.

### Statistics analysis

One-way ANOVA was conducted with SPSS version 16 for data analysis (SPSS, Inc., Chicago, IL, USA). To determine the potential of microRNA-1 and microRNA-221-3p as reliable biomarkers in patients with MI, receiver operator characteristic (ROC) curve analysis was also performed using the expression level of microRNA adjusted for the matching factors. The area under the curve (AUC) was calculated with a 95% confidence interval (CI) and the specificity and sensitivity were identified by numerical integration of each ROC analysis. Correlations between microRNAs expression levels were studied by Pearson’s analysis. The level of significance was considered *p < 0.05* in all calculations. All data were presented in Microsoft Excel version 2010 (Microsoft Corp., Redmond, WA, USA) and XLSTAT version 2016.02.28451 (Addinsoft, Paris, France).

## Results

### Clinical characteristics of patients

The mean age was 55.44±10.8 years and males constituted 60% of the total number of people in the current study. The number of males in the patients and control groups was greater than that of females (male/female=51/49 in the normal group, 70/30 in the primary PCI group and 59/41 in the healthy control group). The mean age of patients in the normal, primary PCI and healthy control groups were 55.24±10.73, 56.68±12.25 and 54.4±9.19 years, respectively. There was no relationship among geographic distribution, weight, blood pressure, gender, age, and diabetes mellitus in patients and controls. Other related risk factors and the laboratory data of patients and healthy controls are shown in Table 1.

**Table 1:** Comparison of quantitative and qualitative variables between patients and healthy controls.

**Variables**	**Normal patients Mean± SD**	**Primary PCI patients Mean± SD**	**Healthy controls Mean± SD**	***P*** **-value**
Age (years)	55.24± 10.73	56.68± 12.25	54.4±9.19	0.001
Gender, male	51(51%)	70(70%)	59(59%)	-
Weight (kg)	80.32±13.16	84.09± 13.16	78.11±9.34	0.001
Systolic Pressure (mmHg)	123.82 ±1.8	146.59± 2.3	124. ±1.7	0.001
Diastolic Pressure (mmHg)	78.67±0.9	82.00± 1.1	79. 5±0.8	0.001
History of heart disease (positive)	34(34%)	40 (40%)	-	-
Family history of heart disease (positive)	50(50%)	64 (64%)	10(10%)	-
Cigarette smoking (positive)	44(44%)	53(53%)	31(31%)	-
Hubble bubble (positive)	21(21%)	23(23%)	9(9%)	-
FBS (mg/dl)	100.50±22.69	100.95± 24.62	89.19±10.08	0.001
CTn I (ng/ml)	0.62± 0.69	4.15±2.29	0.1±0.49	0.001
Urea (mg/dl)	34.13± 13.52	35.88± 13.71	29.76±7.73	0.05
Creatinine (mg/dl)	1.24± 0.70	1.56± 0.54	1±0.17	0.001
C K-MB (U/L)	33.69± 17.52	160.64± 138.85	20.38±33.67	0.001
Blood Sugar (mg/dl)	113.18±47.35	147.19± 55.15	99.03±7.97	0.001
Serum Na (mEq/L)	140.61± 3.02	137.39±17.95	141.07± 3.26	0.01
Serum K (mEq/L)	4.07± 0.4	4.21± 0.45	3.97 ±0.42	0.01
Serum Mg (mg/dl)	2.37± 0.28	2.44± 0.38	2.04±0.19	0.01
Cholesterol (mg/dl)	144.62± 39.86	164.60± 46.3	126.92±31.06	0.025
Triglyceride (mg/dl)	123.87± 64.22	163.99± 46.33	119.19±30.51	0.001
HDL (mg/dl)	49.41±12	42.79± 7.15	70. 54±13.44	0.001
LDL (mg/dl)	104.57± 36.71	111.16± 41.25	70.16±13.93	0.001
SGPT (ALT) (mg/dl)	32.72±21.57	79.57± 115.54	26. 68±7.51	0.001
SGOT(AST) (mg/dl)	31.92± 7.05	79.13±115.69	27.30±8.31	0.001
W.B.C (×1000/mm^3^)	7.94± 2.22	9. 90± 2.93	8.38±1.39	0.001
RDW (%)	13.46± 1.15	13.63± 1.15	13.26±1.86	0.005
MPV (fL)	9.56± 1.12	9.22± 0.88	9.51±1.39	0.006
M.C.V (fL)	89.99± 4.02	89.92± 4.49	90.03±3.54	0.001
M.C.H (Pgm)	29.43± 2.13	30.05± 2.60	29.50±1.82	0.84
M.C.H.C (%)	35.73± 30.36	33.36± 1.33	32.63±1.46	0.001
Platelet (×1000/mm^3^)	231.68± 84.85	247.12± 83.4	287.40±82.77	0.001

Data are presented as the mean with the standard deviation (SD) and count (%)., CTnI = Cardiac troponin I, CK-MB = Creatine kinase-myocardial band, Na^+^ = Sodium, K^+^ =Potassium, TG = Triglyceride, LDL = Low-density lipoprotein, HDL = High density lipoprotein, AST = Aspartate transaminase, ALT = Alanine transaminase, W.B.C = White blood cells, FBS=Fasting blood sugar, MCV=Mean corpuscular volume, MCH=Mean corpuscular hemoglobin, MCHC= Mean corpuscular hemoglobin concentration.

### Circulating microRNA-1 and microRNA-221-3p levels in patients with MI

The circulating levels of microRNA-1and microRNA-221-3p were analyzed using the fold change values in all studied patients and healthy controls. There were significant differences in microRNA-1 and microRNA-221-3p levels between the two patient groups. Statistical analysis revealed up-regulation of microRNA-1 and microRNA-221-3p expression in all patients compared to healthy controls. Expression level of microRNA-221-3p was higher in patients, than healthy controls. For samples obtained from patients, up-regulation of microRNA-221-3p and microRNA-1 occurred in response to heart attack and the levels remained consistent in blood. The microRNA-1 increased 8.5-fold in normal patients and 60.04-fold (*P*  <  0.001) in patients with primary PCI, compared to healthy controls.

**Figure 1:**
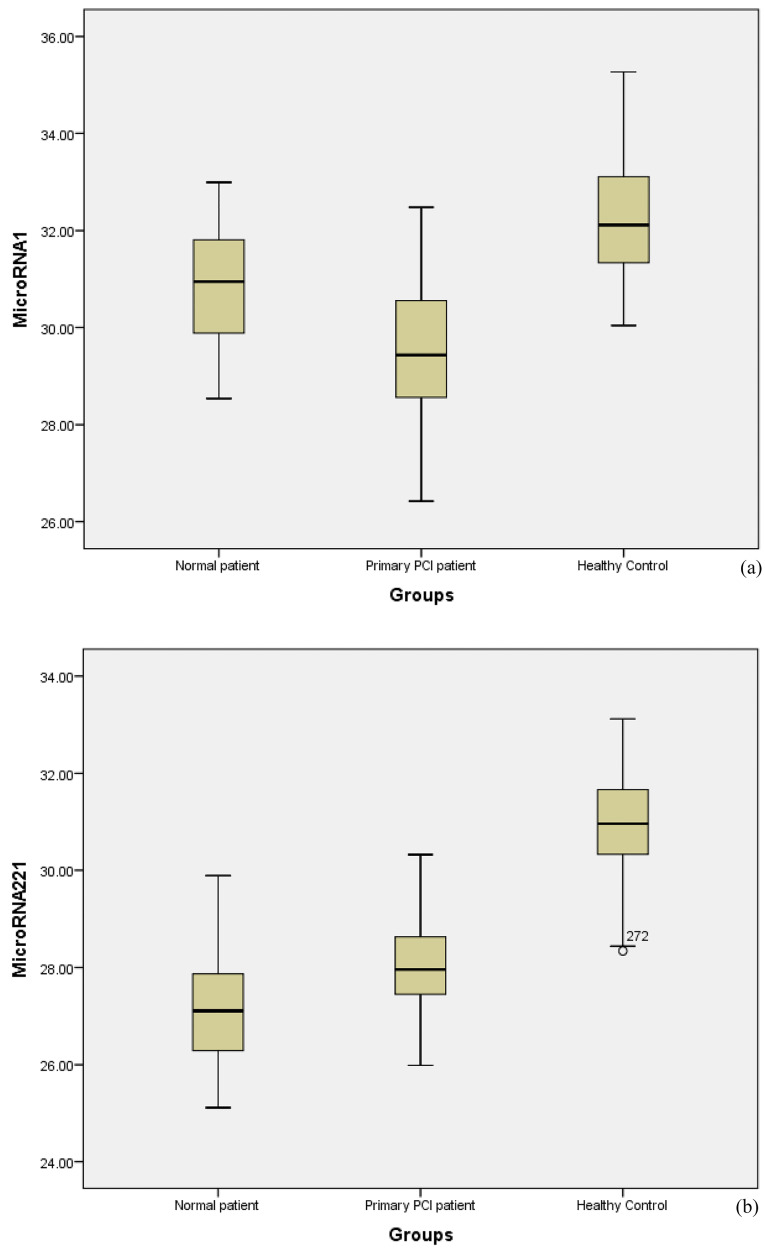
Box plots show the cycle threshold (CT) of microRNA-1(a) and microRNA-221-3p (b) in patients with MI and healthy subjects. CT levels are indicated on the Y-axis and patients and control groups on the X-axis. Increased microRNA-1 indicates the decreased CT values in the two groups of patients compared with the control group.

The microRNA-221-3p increased 31.31-fold in normal patients and 210.66-fold (*P*  <  0.001) in patients with primary PCI compared with healthy controls. The mean of ∆CT, ∆∆CT, and fold changes are shown in Table 2. In patients with MI, microRNA-1 levels were significantly lower than microRNA-221-3p, suggesting an altered expression of microRNA-221-3p in patients with MI. The mean CT was considered for analysis and triplicate reactions were performed for each sample. By comparison, over half of patients with MI (73%) had CT values < 30 and over half of the healthy controls had CT values >30. In the box plots, CT levels of microRNA-1 and microRNA-221-3p are indicated in patients of the normal, primary PCI and healthy control groups (Figure 1).

**Table 2:** Quantitative comparison of delta CT, delta delta CT (ΔΔCT) and fold change in microRNA-1 and microRNA-221-3p between patients and healthy controls.

**Delta Cycle threshold (ΔCT)**	**Normal patients Mean± SD**	**Primary PCI patients Mean± SD**	**Healthy controls Mean± SD**
Mean of Delta CT MicroRNA-1(ΔCT)	5.55±1.49	2.91± 0.92	8.17±1.22
ΔΔCT MicroRNA-1	-2.62± 1.12	-5.26±1.48	29.69±1.253
Fold change 2 ^-ΔΔCT^ MicroRNA-1	8.50± 7.98	60.04±55.48	-
Mean of Delta CT MicroRNA-221-3p(ΔCT)	5.59± 1.66	3±1.16	9.54± 1.49
ΔΔCT MicroRNA-221-3p	-3.94±1.69	-6.53±1.98	8.91±1.41
Fold change 2 ^-ΔΔCT^ MicroRNA-221-3p	31.31±42.49	210.66±36.85	-

Data are revealed as the mean with the standard deviation (SD). Delta CT (ΔCT) =Difference between mean CTs of experimental gene (MicroRNA-1 or MicroRNA-221-3p) and housekeeping gene (microRNA-423-5p) for all groups.

### ROC analysis:The sensitivity and specificity of circulating microRNA-1 and microRNA-221-3p in MI

**Figure 2:**
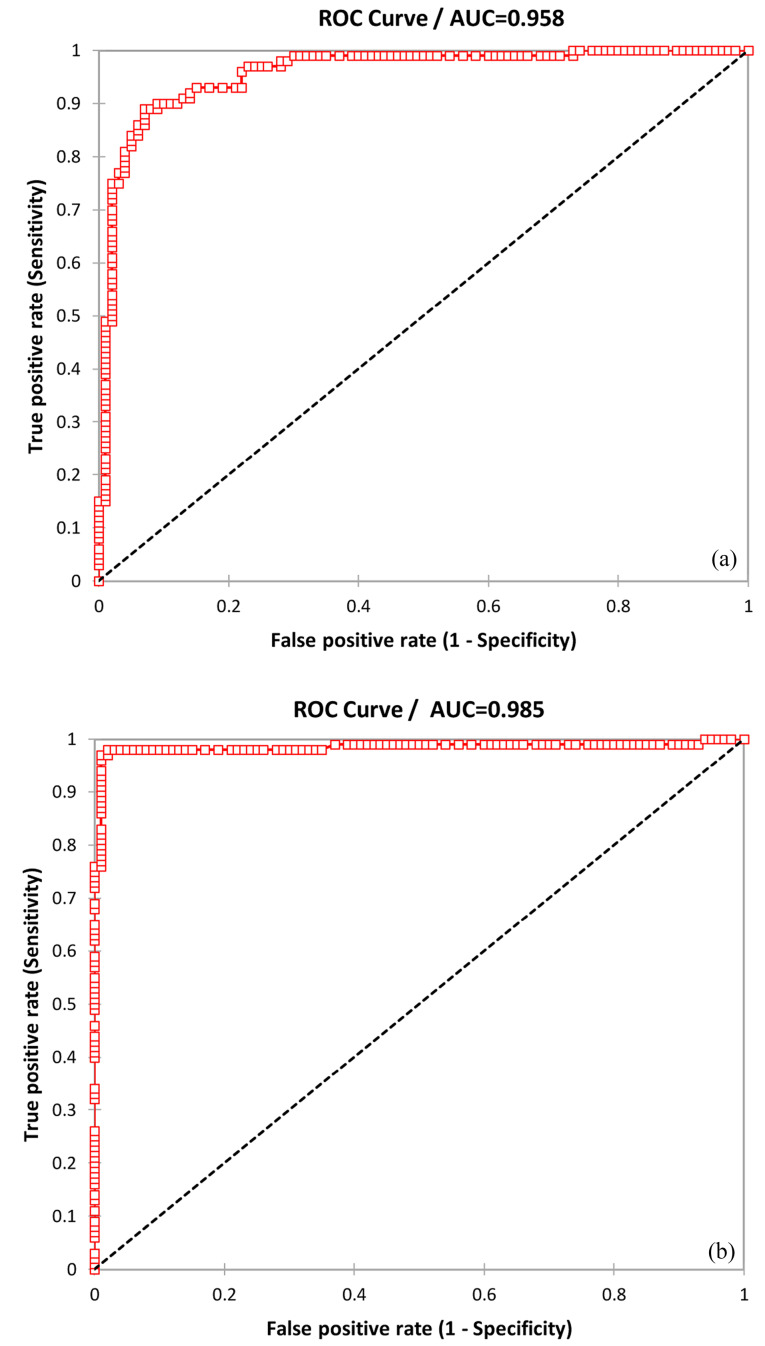
The ROC analysis was performed to investigate microRNA-221-3p expression levels in patients with normal and primary PCI in MI (AUCs 0.958(a) and 0.985(b), respectively).

ROC analysis was performed to evaluate the sensitivity and specificity of circulating microRNA-1 and microRNA-221-3p among patients with AMI. Total sensitivity, specificity, and accuracy of microRNA-221-3p were 0.89, 0.93, and 0.9, respectively; and AUC was 0.958 (95%CI=0.432–0.483, alpha=0.05, *P*  < 0.0001) in normal patients. Also, total sensitivity, specificity, and accuracy of microRNA-221-3p were 0.98, 0.98, and 0.98, respectively; and the AUC was 0.985 (95%CI = 0.467–0.50, alpha=0.05, *P* < 0.0001) in patients with primary PCI (Figure 2).

**Figure 3:**
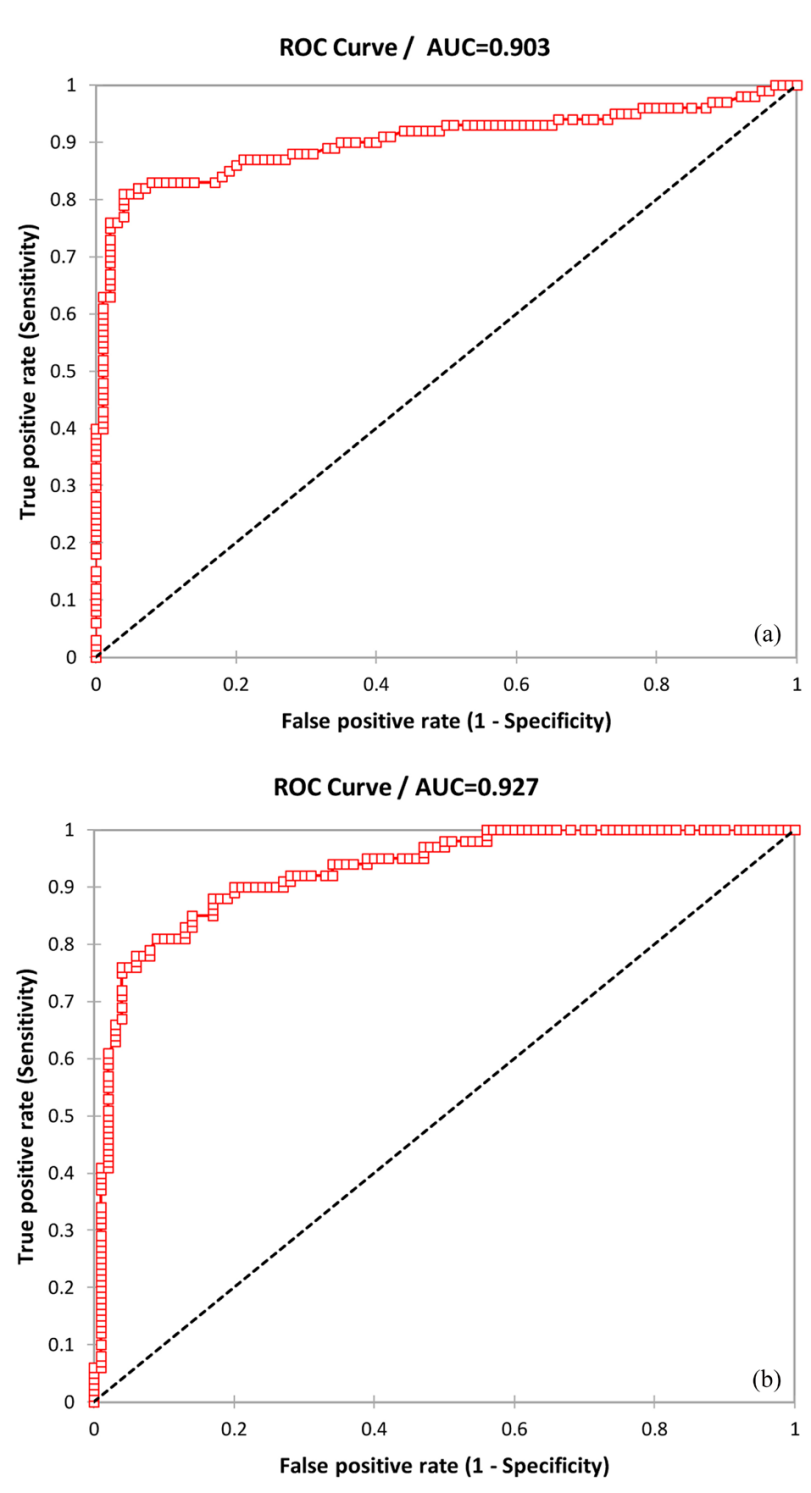
The ROC analysis was performed to investigate microRNA-1 expression levels in patients with normal and primary PCI in MI (AUCs 0.903(a) and 0.927 (b), respectively).

Total sensitivity, specificity, and accuracy of microRNA-1 were 0.81, 0.91, and 0.86, respectively; and AUC was 0.927 (95%CI=0.393–0.461, alpha=0.05, *P*  <  0.0001) in patients with primary PCI. Also, total sensitivity, specificity, and accuracy of microRNA-1 were 0.76, 0.90, and 0.83, respectively; and AUC was 0.903 (95%CI =0.359–0.442, alpha=0.05, *P* < 0.0001) in normal patients (Figure 3).

### Correlation of microRNA-1 and microRNA-221-3p with other cardiac markers

**Figure 4:**
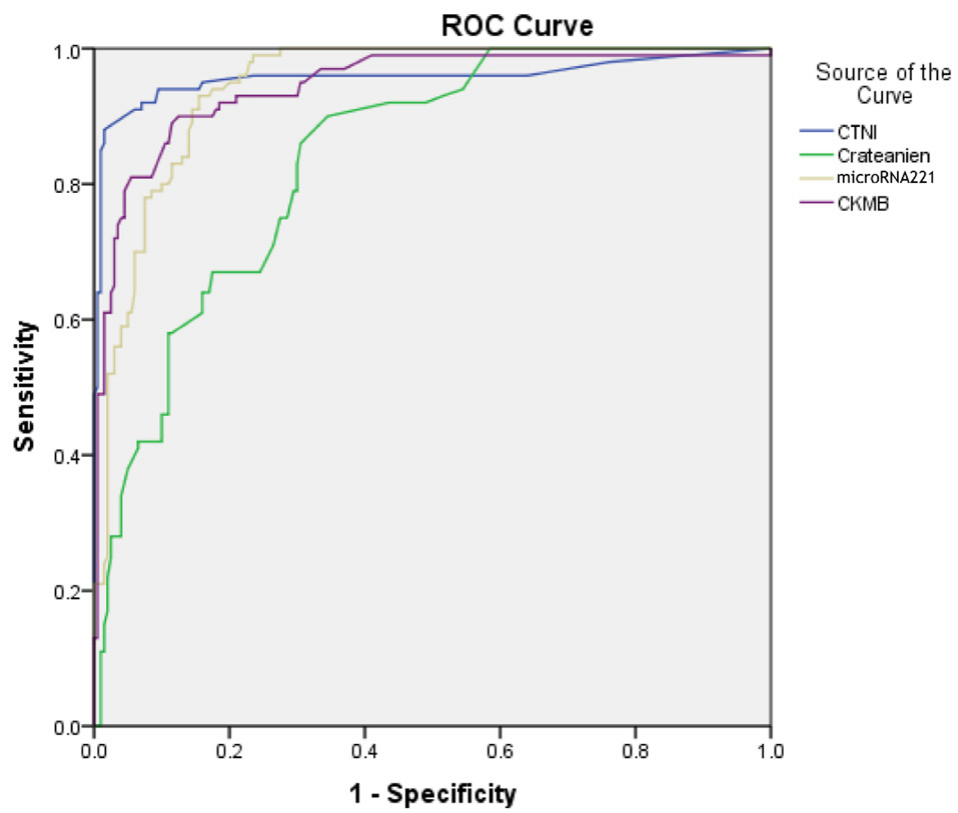
The ROC analysis was performed to investigate microRNA-221-3p expression levels in combination with CK-MB, CTnI, and creatinine in patients with primary PCI

The correlation of the expression level of microRNA-1 and microRNA-221-3p with cardiac markers were analyzed. No significant correlation was detected between microRNA-1 and microRNA-221-3p levels gender, age, weight, systolic and diastolic blood pressure, FBS, serum Na, serum K, WBC, RDW, MPV, MCV, MCH, MCHC levels and platelet count in the studied population. Pearson correlation analysis was performed to evaluate the correlation of microRNA-1 and microRNA-221-3p with other cardiac markers. Furthermore, comparative analyses showed the correlation of microRNA-1 and microRNA-221-3p, with other cardiac markers such as CTnI, creatinine, CK-MB, and *Pearson* correlation of microRNA-221-3p with CTnI Pearson=0.61, creatinine Pearson=0.41, CK-MB Pearson=0.378, alpha=0.05,* (P*  <  0.0001). Correlations of microRNA-1 and microRNA-221-3p with other markers are presented in Tables 3 and 4. Pearson correlations in patients with primary PCI were higher than of normal patients in the two microRNAs. Also, expression levels of the microRNA-1 and microRNA-221-3p were strongly correlated with the concentrations of CTnI and CK-MB, creatinine, FBS, triglyceride, serum Mg, LDL, and HDL levels (negative correlation) and poorly correlated with urea, cholesterol, triglyceride, ALT, AST, and WBC levels in patients with AMI. Interestingly, the combination of these microRNAs led to a much higher AUC (0.99) than that of other markers (AUC= 0.90). The correlation of microRNA-221-3p with plasma levels of CTnI, CK-MB and creatinine were detected in Figure 4.

**Table 3:** Pearson correlations of some variables with microRNA-221-3p fold change.Bold numbers represent significant correlation.

**Variables**	**Pearson**	**R** ^ 2 ^	***P*** ** Values**	**Pearson**	**R** ^ 2 ^	***P*** ** Values**
	**Primary PCI Patients**			**Normal patients**		
CTnI	**0.612**	0.375	** < 0.0001**	**0.189**	0.036	**0.008**
Urea	**0.172**	0.03	**0.015**	**0.145**	0.021	**0.041**
Creatinine	**0.41**	0.168	** < 0.0001**	**0.18**	0.032	**0.011**
CK-MB	**0.378**	0.143	** < 0.0001**	**0.154**	0.024	**0.03**
FBS	**0.473**	0.223	** < 0.0001**	**0.273**	0.075	** < 0.0001**
Serum Na	-0.068	0.005	0.337	0.038	0.001	0.592
Serum K	**0.221**	0.049	**0.002**	-0.004	0.0001	0.958
Serum Mg	**0.461**	0.213	** < 0.0001**	**0.301**	0.09	** < 0.0001**
Cholesterol	**0.171**	0.029	**0.016**	**0.139**	0.019	**0.049**
Triglyceride	**0.227**	0.052	**0.001**	0.043	0.002	0.55
HDL	**-0.552**	0.305	** < 0.0001**	**-0.253**	0.064	**0.0001**
LDL	**0. 377**	0.142	** < 0.0001**	**0.28**	0.079	** < 0.0001**
ALT	**0.18**	0.033	**0.011**	0.031	0.001	0.066
AST	**0.168**	0.028	**0.017**	0.102	0.01	0.149
W.B.C	**0.211**	0.045	**0.003**	-0.019	0.0001	0.794

Pearson’s *r*= Pearson’s Regression, R^2^ = Coefficients of determination

**Table 4:** Pearson correlations of some variables with microRNA-1 fold change.Bold numbers represent significant correlation.

**Variables**	**Pearson**	**R** ^ 2 ^	***P*** ** Values**	**Pearson**	**R** ^ 2 ^	***P*** ** Values**
	**Primary PCI Patients**			**Normal patients**		
CTnI	**0.241**	0.058	**0.001**	**0.2**	0.04	**0.004**
Urea	0.074	0.006	0.296	0.063	0.004	0.378
Creatinine	**0.34**	0.116	** < 0.0001**	0.071	0.005	0.316
CK-MB	**0.312**	0.097	**0.000**	0. 02	0.01	0.152
BS	0.124	0.015	0.08	0.03	0.0001	0.782
Serum Na	**-0.003**	0.0001	0.965	**-0.156**	0.024	0.027
Serum K	0.1	0.01	0.159	0.063	0.004	0.377
Serum Mg	**0.281**	0.079	** < 0.0001**	**0.225**	0.05	**0.001**
Cholesterol	**0.284**	0.081	** < 0.0001**	**0.176**	0.031	**0.013**
Triglyceride	**0.313**	0.098	** < 0.0001**	0. 011	0.0001	**0.874**
HDL	**-0. 327**	0.107	** < 0.0001**	**-0.314**	0.098	**0.0001**
LDL	**0.284**	0.081	** < 0.0001**	0.004	0.0001	0.959
ALT	0.083	0.007	0.243	0.008	0.0001	0.911
AST	0.101	0.01	0.155	0.011	0.0001	0.874
W.B.C	0.058	0.003	0.417	-0.053	0.003	0.453

Pearson’s *r*= Pearson’s Regression, R^2^ = Coefficients of determination

## Discussion

MI is strongly associated with psychological changes in cardiovascular system and especially increases by aging.^14^ The current diagnostic methods for MI focus on cardiac laboratory tests; a large number of the affected population suffer from late diagnosis of the medication and about 50% of people do not have any cardiac pain symptoms. Although troponin and other tests, as gold standard measures, can definitely help to MI diagnosis, these cardiac markers can be tracked in blood within 3-8 hours after MI with a peak at 12 to 24 hours, and some patients may be discharged from the hospital.^8^ This is the first study to investigate the potential association between microRNA-1 and microRNA-221-3p in MI risk. Furthermore, obtaining blood samples from hospitalized patients is faster and easier than other samples. Recently, novel rule-in and rule-out strategies are designed, which can detect cardiac markers of MI during in the first hour of onset.^15^ Thus, cardiologists show more interest to the early detection of MI in choosing effective prescriptions. Recent studies highlighted the role of high microRNAs level in cell-to-cell communication and cardiac muscle-specific function based on the results of blood or other biofluids tests.^16,17^ It is also predicted that microRNA changes such as up-regulation or down-regulation occur at much earlier stages of disease development, while the accurate diagnosis of MI is a target for prognosis and treatment of patients.^18,19^

In the current study, the predictive value of circulating microRNAs, including microRNA-1 and microRNA-221-3p, as informative markers for MI, was determined in 200 patients on their presentation to the emergency ward of a cardiovascular medical center using a 300-μl of whole blood sample. Both of the tested microRNAs significantly increased in patients with MI, even in cases whose symptoms were manifested within 1-12 hours or the ones with initially negative CTnI. The findings of the current study were consistent with Li et al*.,* and Ai et al., that reported that the level of microRNA-1 significantly increased during acute MI.^20-22^

To determine the diagnostic value of microRNA-1 and microRNA-221-3p, ROC curve analyses were performed. Diagnostic value of microRNA-1 and microRNA-221-3p significantly improved in all patients with MI, when were added to CTnI with an AUC of 0.99. Interestingly, these microRNAs were strong predictors of MI independent of clinical variables including patient’s history and other cardiovascular risk factors. Moreover, the combination of these microRNAs led to a much higher AUC (0.99) than either microRNA alone for the diagnosis. The current study indicated that microRNA-1 and microRNA-221-3p could add predictive power to the established standard for MI. MicroRNA-1 and microRNA-221-3p had the highest AUC in the primary PCI groups. The level of microRNA-221-3p was significantly higher in patients with primary PCI than normal patients. MicroRNA-221-3p had a sensitivity of 98% and a specificity of 98%, while microRNA-1 had a sensitivity of 81% and a specificity of 91% in patients with primary PCI. These microRNAs (with the highest AUC) significantly improved the diagnostic potential of CTnI and other cardiac markers. Individually, both of the AUCs exceeded the value of 0.90, but microRNA-221-3p achieved an AUC of 0.985. Corresponding ROC analysis revealed the high accuracy of microRNA-221-3p in differentiating MIs with more than 50% coronary artery stenosis.

The current study suggested that microRNA-221-3p probably improves diagnostics for patients seeking medical care after acute chest pain. These increase in microRNAs were accompanied by increase other cardiac markers and artery status. Moreover, microRNA-1 and microRNA-221-3p levels increased in patients with primary PCI compared to normal patients. MicroRNA-221-3p significantly up-regulated 210-fold in patients with primary PCI, while microRNA-221-3p up-regulated 31.31-fold in normal patients compared to healthy controls; for better explanation, up-regulation of microRNA-221-3p occurred in response to heart attack or damage to cardiac tissue and the levels remained consistent in blood, compared to microRNA-1 in patients.

MicroRNAs are stable in blood circulation and down/up regulation of microRNAs can directly reflect cardiovascular disease status, and can be utilized as biomarkers in the early detection of MIs, inflammation, apoptosis, angiogenesis, and atherosclerosis. They are useful markers for the diagnosis of multiple diseases.^23,24^ Significant differences were observed in clinical coronary artery status by coronary angiography (CAG) and poor correlation of these microRNAs with cholesterol, triglyceride, and WBC levels in patients with MI. According to these results, microRNA-221-3p seemed more sensitive and specific than circulating microRNA-1 in early detection primary PCI and normal patients, similar to the cardiac-specific blood test and had a pivotal role in regulating cholesterol and triglyceride metabolism. The current study verified the diagnostic values of microRNA-1 and microRNA-221-3p in the early detection of damage to cardiac tissue in comparison with the established marker cTnI. In patients with MI the effect of inflammation on epicardial artery and severity of luminal narrowing is established. The differential expression of microRNA-221 was associated with the severity of MI and reflected the degree of cardiac artery stenosis in the PCI groups.

It was found that microRNA-221-3p might serve as a biomarker to diagnose MI, discriminate between normal and primary PCI patients, and facilitate the management of heart disease. Due to its high sensitivity and specificity, it is proposed to use microRNA-221-3p as a biomarker to detect MI in patients with primary PCI in order to reduce mortality in the affected patients.^18,25^

At present, microRNAs offer additional prognostic values in combination with other cardiac markers. The current single–center study was performed on a limited number of patients, which was far from the cellular and molecular research centers in Iran. Moreover, microRNA measurement was very time-consuming. The microRNA-1 and microRNA-221 levels were determined in patients with AMI on their first presentation. For this reason, multicenter studies with easily available, large-scale samples are required to allow broad use of microRNAs as useful approach in patients with MI.

## Conclusion

Circulatory microRNA-1 and microRNA-221 levels are in correlation with increase in other cardiac tests that are changed in cardiovascular disease. The potential of microRNA-1 and microRNA-221 as non-invasive biomarkers are important for the maintenance of MI risks and predict the risk of narrowing or blockage arteries. These changes in microRNAs could be used the new trends in the development of microRNA therapeutic strategies in clinical practices. The use of anti-microRNAs could be considered as suitable tool in cardiovascular regenerative medicine and research.

## Ethical Issues

This study was performed in accordance with WMA Declaration of Helsinki – Ethical Principles for Medical and approved by the Committee for Ethical Consent of Urmia University of medical sciences under the number IR. UMSU.REC.1395. 252.

## Conflict of Interest

All authors declare no conflict of interests regarding this manuscript.

## Acknowledgments

We thank Shabnam Ashena for the helpful 52 samples collecting form Hospital.

## Funding

This article was financially supported by the Cellular and Molecular Research Center of Urmia University of Medical Sciences, Urmia, Iran (Grant Number 2051).
